# Maternal post-natal tobacco use and current parental tobacco use is associated with higher body mass index in children and adolescents: an international cross-sectional study

**DOI:** 10.1186/s12887-015-0538-x

**Published:** 2015-12-24

**Authors:** Irene Braithwaite, Alistair W. Stewart, Robert J. Hancox, Richard Beasley, Rinki Murphy, Edwin A. Mitchell, the ISAAC Phase Three Study Group

**Affiliations:** Medical Research Institute of New Zealand, Private Bag 7902, Newtown, Wellington 6242 New Zealand; School of Population Health, The University of Auckland, Private Bag 92019, Auckland, 1142 New Zealand; Department of Preventive & Social Medicine, Dunedin School of Medicine, University of Otago, Dunedin, 9016 New Zealand; Department of Medicine, Faculty of Medicine and Health Sciences, The University of Auckland, Auckland, New Zealand; Department of Paediatrics: Child and Youth Health, Faculty of Medicine and Health Sciences, The University of Auckland, Auckland, New Zealand

**Keywords:** BMI, Tobacco use, Smoking, International, Parental smoking, Body mass index, Child, Adolescent, Obesity, Overweight

## Abstract

**Background:**

We investigated whether maternal smoking in the first year of life or any current parental smoking is associated with childhood or adolescent body mass index (BMI).

**Methods:**

Secondary analysis of data from a multi-centre, multi-country, cross-sectional study (ISAAC Phase Three). Parents/guardians of children aged 6–7 years completed questionnaires about their children’s current height and weight, whether their mother smoked in the first year of the child’s life and current smoking habits of both parents. Adolescents aged 13–14 years completed questionnaires about their height, weight and current parental smoking habits. A general linear mixed model was used to determine the association between BMI and parental smoking.

**Results:**

77,192 children (18 countries) and 194 727 adolescents (35 countries) were included. The BMI of children exposed to maternal smoking during their first year of life was 0.11 kg/m^2^ greater than those who were not (*P* = 0.0033). The BMI of children of currently smoking parents was greater than those with non-smoking parents (maternal smoking: +0.08 kg/m^2^ (*P* = 0.0131), paternal smoking: +0.10 kg/m^2^ (*P* < 0.0001)). The BMI of female adolescents exposed to maternal or paternal smoking was 0.23 kg/m^2^ and 0.09 kg/m^2^ greater respectively than those who were not exposed (*P* < 0.0001). The BMI of male adolescents was greater with maternal smoking exposure, but not paternal smoking (0.19 kg/m^2^, *P* < 0.0001 and 0.03 kg/m^2^, *P* = 0.14 respectively).

**Conclusion:**

Parental smoking is associated with higher BMI values in children and adolescents. Whether this is due to a direct effect of parental smoking or to confounding cannot be established from this observational study.

**Electronic supplementary material:**

The online version of this article (doi:10.1186/s12887-015-0538-x) contains supplementary material, which is available to authorized users.

## Background

The rising prevalence of childhood obesity is marked [1, 2]. Concerns about the future health implications of obesity in childhood are well documented [3, 4]. This problem has been identified in low and middle income countries as well as affluent countries [5–7].

Potential contributors to childhood obesity are multiple and complex. Maternal smoking during pregnancy has been identified as a risk factor for low birth weight and small-for-gestational-age infants [8, 9] and as a likely contributor to increased body mass index (BMI) in later life [10, 11].

A number of mechanisms for the association between maternal smoking in pregnancy and increased offspring BMI have been proposed. Nicotine and carbon monoxide exposure have been shown to cause placental vasoconstriction and foetal hypoxaemia, leading to low initial birthweight. [12] Low birthweight babies have been shown to experience rapid catch-up growth in infancy and to have higher risk for overweight and obesity in adolescence and adulthood [13–15]. It has been hypothesised that in infants exposed to intra-uterine nicotine, this phenomenon may be due to changes in the hypothalamic-pituitary axis, affecting satiety and impulse control [16]. Alternatively, the association between maternal smoking in pregnancy and increased offspring BMI may be due to confounding of other lifestyle habits of smoking parents.

Associations between higher BMIs in children and currently smoking parents have been demonstrated in previous studies [17–24] that have predominantly been limited to small cohorts within countries. A number of these have demonstrated an association between maternal and/or paternal smoking independently of maternal smoking before or during pregnancy. Women who smoke during pregnancy have different sociodemographic and anthropometric characteristics than non-smokers and it is likely that children who live in smoking households also have different dietary patterns than those from non-smoking households [25]. In this study we have the opportunity to assess the association between current parental smoking and BMI of a large number of children and adolescents from a range of countries, adjusting for fast food consumption.

The International Study of Asthma and Allergies in Childhood (ISAAC) Phase Three is a multi-national multi-centre study that has previously collected data on heights and weights of children aged 6–7 years and 13–14 years as well as their exposure to parental smoking at various time points in their lives. Although originally designed to measure time trends in the prevalence and severity of asthma, rhinoconjunctivitis and eczema and to explore the relationship between lifestyle, other putative risk factors and the development of asthma and allergies [26], it has provided us with the opportunity to explore the relationship between lifestyle or environmental factors, such as parental smoking, and BMI.

In ISAAC Phase Three information on parental smoking was gathered through an environmental questionnaire (EQ) that was optional for parents of children and adolescents themselves to answer. The EQ also asked for information on fast food consumption of participants, which we took into account as a confounding variable given the sociodemographic patterning of unhealthy lifestyles [25].

Here we present analyses of exposure to environmental tobacco exposure and BMI of children (aged 6 to 7 years) and adolescents (aged 13 to 14 years). We hypothesised that maternal smoking in the first year of life would be associated with a greater BMI in children, and that there would be a similar association between current maternal and/or paternal smoking and BMI of children and adolescents.

## Methods

This study is a secondary analysis of the data gathered during the ISAAC Phase Three study. Permission was granted by the ISAAC Steering Committee to access the data. ISAAC is a multicentre, multi-country, multiphase, cross-sectional study investigating the prevalence of the symptoms of asthma, rhinoconjunctivitis and eczema, and the role of risk factors, as previously described [26]. ISAAC Phase Three included 116 sites that had originally participated in ISAAC Phase One and 168 sites that were new to Phase Three. A minimum of 10 schools were randomly sampled within pre-defined geographic areas (centres). Participants (13–14 year olds and 6–7 year olds) were selected from within those schools depending on the local situation; either the grade, level or year where classes with the most children in the age ranges were selected, or by age group, where only children within that age group, regardless of grade, level or year were selected. ISAAC Phase Three used the Phase One standardised core questionnaire on symptoms of asthma, rhinoconjunctivitis and eczema, and included an optional environmental questionnaire (EQ) to collect potential risk factor data including height, weight, and parental smoking. The EQ was developed by the ISAAC steering committee to assess potential risk factors for the development of asthma in children that had been identified in previous research. Where possible, questions previously published in the literature were replicated in the EQ, otherwise the questions were developed by the ISAAC steering committee. The EQ was piloted in New Zealand, Latin America, French Speaking Africa and the Asia-Pacific regions and was optional for all participating centres. Instructions were provided in the event centres wished to translate it into the local language. Adolescents self-completed their questionnaires while at school and children were sent home with questionnaires for their parents or guardians to complete. A participation rate of 90 % was targeted. Where participation rates were below 90 % for adolescents, a second visit was carried out to include those that were absent when the EQ was originally answered. The EQ was issued a number of times to parents of the 6–7 year olds in case the children were away from school due to ill health. The questionnaires are on the ISAAC website [27].

### Main outcome variable - body mass index

Height and weight were reported by the parents of the children, and were self-reported by adolescents. In some centres, each subject’s height and weight were measured objectively; there were no standardised or specific instructions for doing this. BMI was calculated (weight (kg)/height (m)^2^). We subsequently adjusted for whether heights and weights were measured or reported in each centre.

### Explanatory variables

Parental smoking of study participants was assessed using the following questions:

For children:

1. Does your child’s mother (or female guardian) smoke cigarettes?
  - If yes, how many cigarettes does the child’s mother (or female guardian) smoke each day?
2. Does your child’s father (or male guardian) smoke cigarettes?
  - If yes, about how many cigarettes does the child’s father (or male guardian) smoke each day?
3. Did your child’s mother (or female guardian) smoke cigarettes during your child’s first year of life?

For adolescents:

1. Does your mother (or female guardian) smoke cigarettes?
2. Does your father (or male guardian) smoke cigarettes?

Each explanatory variable was examined separately for both age groups. To assess the presence of a dose–response relationship in the children, the number of cigarettes smoked per day by the parents were categorised for the purpose of analysis as 0, 1–9, 10–19, 20–29, and 30 or more.

Fast food consumption was assessed by parents of children and adolescents reporting their weekly consumption of ‘fast food’/‘burgers’ over the previous 12 months, categorised in the questionnaire as’never or occasionally’, ‘once or twice per week’, and ‘three or more times per week’.

Country GNI was based on the 2006 World Bank of Gross National Income by country. The World Bank categories of high-, high middle-, low middle-, and low-income countries were dichotomised into high income (high- plus high middle-income) and low income (low middle- plus low-income) categories.

### Participants

For the children, data which included heights, weights and parental smoking variables were submitted from 73 centres in 32 countries (214 706 subjects). For the adolescents, data were submitted from 122 centres in 53 countries (362 091 subjects).

Centres that provided >70 % data for current maternal smoking were included in our analyses. Some of these centres did not gather data on current paternal smoking or in the case of children, maternal smoking in the first year of life. Individuals without complete age, sex, fast food consumption, height or weight data were excluded.

### Data cleaning

To preserve as much BMI data as possible, but also to eliminate likely erroneous data, we applied the following thresholds:

- For children in each centre, those in the top and bottom 0.5 % of weights and heights (*n* = 1,391), and those with heights less than 1.0 metre were excluded (*n* = 346). Children with BMI less than 9 kg/m^2^ and greater than 40 kg/m^2^ were excluded (*n* = 215).
- For adolescents in each centre, those in the top and bottom 0.5 % of weights and heights (*n* = 3,712), and those with heights less than 1.25 m were excluded (*n* = 904). Adolescents with BMI less than 10 kg/m^2^ and greater than 45 kg/m^2^ were excluded (*n* = 360).

Following sequential application of the exclusion and data cleaning criteria described above, 77 192 children (31 centres/18 countries) and 194 727 adolescents (72 centres/35 countries) were included in the final analysis (Fig. 1). 147 274 adolescents from 55 centres provided self-reported height and weight while 47 453 adolescents from 17 centres provided measured heights and weights (Additional file 1: Figure S1).

### Statistical analysis

BMI was assessed separately for each age group using a general linear mixed model with centre as a random effect and GNI for each country, the individual’s age, sex, measurement type, fast food consumption, and each parental smoking variable as fixed effects. The BMI values reported are the modelled means for those who had no exposure to either parent smoking for all ages in the children and adolescent’s groups respectively.

Because of an interaction found between paternal smoking and GNI, separate estimates were made for paternal smoking at each GNI level for both children and adolescents. Further analyses were undertaken separately for male and female adolescents and those whose heights and weights were objectively measured due to interactions found between sex and current maternal smoking, sex and current paternal smoking, measurement type and current maternal smoking, and measurement type and current paternal smoking.

## Results

For the children, the basic characteristics of each centre are shown in Additional file 2: Table S1, and for the adolescents, basic characteristics are shown in Additional file 3: Table S2.

### Exposure to parental smoking

In the children, 9.9 % had been exposed to maternal smoking in their first year of life (Additional file 4: Figure S2a). 43.1 % of children were exposed to some kind of current parental smoking (10.4 % both parents, 4.6 % maternal smoking only and 28.1 % paternal smoking only) (Additional file 4: Figure S2b).

44.4 % of adolescents reported exposure to current parental smoking (12.4 % to both parents, 6.9 % to maternal smoking only, and 25.2 % to paternal smoking only) (Additional file 4: Figure S2c).

### Exposure to parental smoking and BMI

In both age groups GNI and fast food variables showed a significant association with BMI but did not have any influence on the smoking-BMI associations.

### Children

Figure 1a shows the difference in BMI (kg/m^2^) between children not exposed to parental smoking at any time point and those exposed to maternal smoking in the first year of life, current maternal smoking and current paternal smoking in each centre, with countries grouped into high and low GNI categories .

There were no interactions between any maternal smoking variables, current paternal smoking, measurement type, sex or age. Because of a significant interaction found between GNI and paternal smoking, estimates are given for paternal smoking by GNI category.

The estimated mean BMIs in children not exposed to parental smoking at all were 14.4 and 14.7 kg/m^2^ for ages 6 and 7 respectively. After controlling for country GNI, centre, individual fast food usage, age and measurement type, there was an association between exposure to parental smoking at any time and BMI (Table 1). In high GNI countries children of smoking fathers had larger BMIs, than those with non-smoking fathers, while in low GNI countries children of smoking fathers had smaller BMIs than those of non-smoking fathers (Table 1).

**Table 1:** Association between parental smoking and BMI of study participants (+/− kg/m^2^, (SE) and *P* value)

No exposure to parental smoking		Mother smoked 1^st^ year of life	Mother currently smokes	Father currently smokes	Father currently smokes
				High GNI countries	Low GNI countries
Children (*N* = 77 192)	14.66^a^	+0.11 (0.04) *P* = 0.002	+0.07 (0.03) *P* = 0.03	+0.15 (0.02) *P* < 0.0001	−0.14 (0.05) *P* = 0.004
Adolescent Females (*N* = 98 238)	19.72^a^	N/A	+0.22 (0.03) *P* < 0.0001	+0.18 (0.03) *P* < 0.0001	−0.05 (0.04) *P* = 0.17
Adolescent Males (*N* = 96 489)	19.78^a^	N/A	+0.18 (0.03) *P* < 0.0001	+0.06 (0.03) *P* = 0.04	−0.03 (0.04) *P* = 0.48

^a^Estimated BMIs for boys aged 7 years and adolescents aged 14 years. Associations stated are additive

There was a dose response relationship between the number of cigarettes smoked daily by each parent and the BMI of the child (Table 2).

**Table 2:** Association between the number of cigarettes smoked daily by parents and BMI of the children, compared to BMI of children whose parents do not smoke (+/− kg/m^2^, (SE) and *P* value

Number of cigarettes smoked daily	None	1–9	10–19	20–29	30+
Maternal *P* < 0.0001	-	+0.04 kg/m^2^ (0.04)	+0.20 kg/m^2^ (0.04)	+0.35 kg/m^2^ (0.05)	+0.51 kg/m^2^ (0.14)
Paternal *P* < 0.0001	-	+0.06 kg/m^2^ (0.03)	+0.13 kg/m^2^ (0.03)	+0.19 kg/m^2^ (0.03)	+0.34 kg/m^2^ (0.06)

### Adolescents

Figure 1b shows the difference in BMI (kg/m^2^) between adolescents with no exposure to current parental smoking and those with current maternal or paternal smoking in each centre.

Because of significant interactions found between sex and maternal smoking, sex and paternal smoking, measurement type and both maternal and paternal smoking, analyses were done separately for each sex, and then using measured height and weight data only. There was also an interaction between GNI and paternal smoking, so estimates are given for paternal smoking by GNI.

Data were available for 98 238 females. For those not exposed to parental smoking, estimated mean BMIs were 19.32 and 19.72 kg/m^2^ for ages 13 and 14 respectively. After controlling for country GNI, centre, individual fast food consumption, age and measurement type, there was an association between BMI and both maternal (+0.23 kg/m^2^) and paternal (High GNI +0.18 kg/m2 and Low GNI −0.05 kg/m2) smoking (Table 1).

When analyses were restricted to those adolescent females who had measured height and weight data (*N* = 25 675), there still appeared to be a tendency towards a higher BMI with maternal smoking, (+0.11 kg/m^2^: SE 0.06, *P* = 0.06), but no association between paternal smoking and BMI (−0.03 kg/m^2^: SE 0.05, *P* = 0.54).

Data were available for 96 489 males. For those not exposed to parental smoking, the estimated mean BMIs were 19.51 and 19.78 kg/m^2^ for ages 13, and 14 respectively. After controlling for country GNI, centre, individual fast food consumption, age and measurement type, there was an association between BMI and maternal smoking (0.19 kg/m^2^), but not paternal smoking (Table 1).

When analysis was restricted to those adolescent males that supplied measured height and weight data (*N* = 21 778) there was no significant association between maternal or paternal smoking and BMI (maternal smoking: (−0.02 kg/m^2^: SE 0.06, *P* = 0.80), paternal smoking: (−0.01 kg/m^2^: SE 0.05, *P* = 0.82)).

## Discussion

In this study which included populations with wide variation in the social patterning of smoking, we have demonstrated an association between maternal smoking in the first year of life and a greater BMI in children by the age 6–7 years. We have also shown independent but additive associations between current maternal or paternal smoking and children’s BMI at age 6–7. Children whose mother smoked in their first year of life and who had both parents currently smoking had BMIs that were on average 0.29 kg/m^2^ greater than children who had no exposure to parental smoking at all. We also found a dose–response between the number of cigarettes smoked daily by each parent and the BMI of the child. In adolescents we found adolescent females had a larger BMI if their mother or father smoked, and males had a larger BMI if their mothers smoked.

Our findings in children are consistent with those of Raum et al. who found a greater BMI at the age of 6 in offspring of mothers who smoked both in the first year of life and currently, independent of maternal smoking before or during pregnancy [17]. Kaufman-Shriqui and colleagues [28] reported an association between current maternal smoking and overweight or obesity in a small sample of lower socioeconomic Israeli children aged 4 to 7 years. Florath and colleagues [23] demonstrated a significant association between current maternal smoking and the BMI of 8 year old German children, and a slightly larger association between paternal smoking and BMI in the same sample. Conversely, Toschke and colleagues [29] evaluated associations between maternal smoking patterns pre- and post-pregnancy and the BMI of children aged from 5 to 7 years. They found an association between maternal smoking in pregnancy and obesity in children, but no association with smoking after pregnancy, concluding that intrauterine exposure to tobacco smoking was instrumental in the association. In a study from Japan, Oyama and colleagues [30] also concluded that while smoking during pregnancy was independently associated with rapid weight gain between one and 18 months of age, but daily current smoking by the mother was not.

We did not have any data available on maternal smoking during pregnancy, so were not able to test whether the associations between childhood BMI and current parental smoking in our sample were independent of this variable. It is likely that maternal smoking in the first year of an infant’s life is associated with maternal smoking during pregnancy and some of the association between maternal smoking in the first year of life and the child’s BMI may be explained in this way. However, the independent association and dose response effect we have seen between current maternal and paternal smoking and BMI of children at the age of 6 years supports causal inference.

In our assessment of adolescent data, the association between current parental smoking and BMI was less clear. There was a greater BMI in adolescent females with either parent smoking, but adolescent males had a greater BMI when their mother smoked, not when their father smoked. When analysis was restricted to adolescents that supplied measured heights and weights only, the effect sizes were smaller and not statistically significant. Few studies have previously explored this association. Associations between parental smoking and BMI have been demonstrated in Taiwanese 9 to 14 year olds by Chen and colleagues [31], and in Israel Huerta et al [24] have shown that parental smoking is an independent risk factor for overweight and severe overweight in 8–13 year old offspring, and that there was a dose–response relationship between the number of parental smokers and the risk of overweight. The estimated effect on BMI in our sample was small at an individual level (additive effect of up to 0.29 kg/m^2^ in children, 0.18 kg/m^2^ and 0.22 kg/m^2^ in male and female adolescents respectively). However, given the long term consequences of childhood overweight and obesity, even a small change in the mean BMI within a population could be of major public health significance.

This study has also shown that a large proportion of children and adolescents report parental cigarette smoking despite its well-known associations with childhood illnesses. Current maternal smoking was reported in 15 % and 20 % of children and adolescents respectively. 39 % of children and 38 % adolescents in our sample reported current paternal smoking. Given that paternal smoking was independently associated with an increased BMI in children, the high proportion of children potentially exposed to paternal smoking is of concern. Our finding that children and adolescents of smoking fathers in high GNI countries had larger BMIs than those of non-smoking fathers might be consistent with the observation that both smoking behaviours and obesity have tended to become more concentrated in lower socioeconomic groups within high GNI countries [32, 33], although we did not have each individuals’ socioeconomic information to confirm this theory. The lower BMI found in children of smoking fathers in low GNI countries is more difficult to explain, but with only a small number of centres and participants contributing to the analysis, this result may be spurious.

Because of the observational nature of this study, we cannot determine that parental smoking is the cause of an elevated BMI. It is possible that smoking may be a marker of other factors that influence BMI such as socioeconomic status, dietary factors, maternal smoking during pregnancy, physical activity or inactivity or whether the adolescents themselves smoked cigarettes. Of these possible confounders, we were able to adjust for fast food as a marker of obesogenic dietary habits which did not alter the associations.

The mechanisms for an association between childhood exposure to parental smoking and BMI are not yet identified. It is possible that parental smoking is reflective of an unhealthy lifestyle associated with other factors that lead to an increase in childhood BMI [30, 34], or possibly parents may smoke with the perception that this is helping to control their own weight, and so are less vigilant about family diet. The weaker association between BMI and parental smoking in adolescence may reflect increasing independence of the adolescent from the household, thus they are less exposed to parental smoking and associated lifestyle factors.

## Strengths and limitations

The major strengths of this study are its size and multicentre structure, with 194 727 adolescents from 35 countries and 77 192 children from 18 countries. Many of the centres were from middle and low income countries from which data on the association between parental smoking and BMI have not previously been reported.

The main limitation to this study is the observational design which allows identification of associations, but not of temporal sequence or causality. The assessments were undertaken by questionnaire leading to errors in the parent-reported weights of their children and self-reported weights of the adolescents. Parents may also have misreported their own smoking levels. Such misclassifications are likely to have reduced any effect towards a null hypothesis. For centres that objectively measured heights and weights, there were no standardised instructions for doing this.

ISAAC comprised a self-selected group of centres without intent to represent any population. The subset of ISAAC Centres that then decided to utilise the Environmental Questionnaire is also a self-selected group. This paper outlines the findings only in the sample that participated in the study, thus there is the possibility that these results are not representative of the population. Although we were able to adjust the analysis for GNI, centre, and each subjects’ fast food consumption, BMI measurement type, and sex in our analysis, we have no data on maternal smoking during pregnancy, individual socioeconomic status, parental BMI, or whether adolescents themselves smoked, all potentially affecting young peoples’ BMI [28, 31, 35].

## Conclusions

This study has demonstrated an association between exposure to maternal smoking in the first year of life and greater BMIs of 6–7 year old children and that current maternal or paternal smoking may pose a risk of similar magnitude, with a dose response effect. Exposure to current maternal or paternal smoking is associated with greater BMIs in adolescent females, while only maternal smoking is associated with greater BMIs in adolescent males. As for all observational studies, causality cannot be proven, but the findings raise the possibility that current parental smoking may contribute to overweight and obesity in childhood.

## Additional files

Additional file 1: Figure S1. Flow of subjects through study. Children are represented in panel (a) and adolescents in panel (b). (TIFF 3798 kb) Additional file 2: Table S1. basic characteristics of contributing centres for 6–7 year old children, including association between parental smoking and BMI (+/- kg/m^2^, (SE)) of participants in each centre. (DOC 73 kb) Additional file 3: Table S2. basic characteristics of contributing centres for adolescents, including association between parental smoking and BMI (+/- kg/m^2^, (SE)) of participants in each centre. (DOC 122 kb) Additional file 4: Figure S2. Reported exposure of study subjects to parental smoking. Panel (a) shows the proportion of 6–7 year olds exposed maternal smoking in their first year of life, panel (b) shows the proportion of 6–7 year olds exposed to any current parental smoking, and panel (c) shows the proportion of adolescents exposed to any current parental smoking^ii^. (TIFF 8254 kb)
