# Supplementary figures and images for: Maternal post-natal tobacco use and current parental tobacco use is associated with higher body mass index in children and adolescents: an international cross-sectional study
Source: BMC Pediatr. 2015 Dec 24;15:220. doi: 10.1186/s12887-015-0538-x (PMC4690230; doi:10.1186/s12887-015-0538-x)

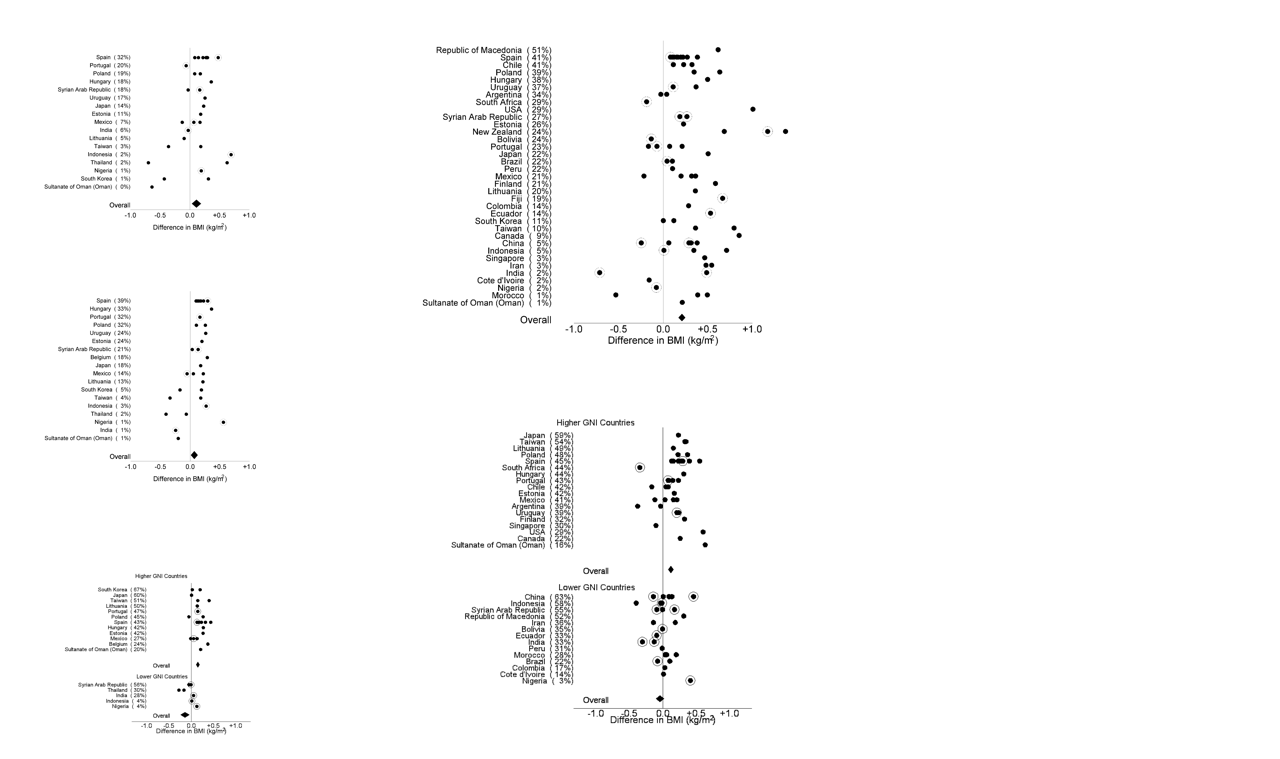

Supplement: Additional file 1: Figure S1. — Flow of subjects through study. Children are represented in panel (a) and adolescents in panel (b). (TIFF 3798 kb) [file 12887_2015_538_MOESM1_ESM.tiff]

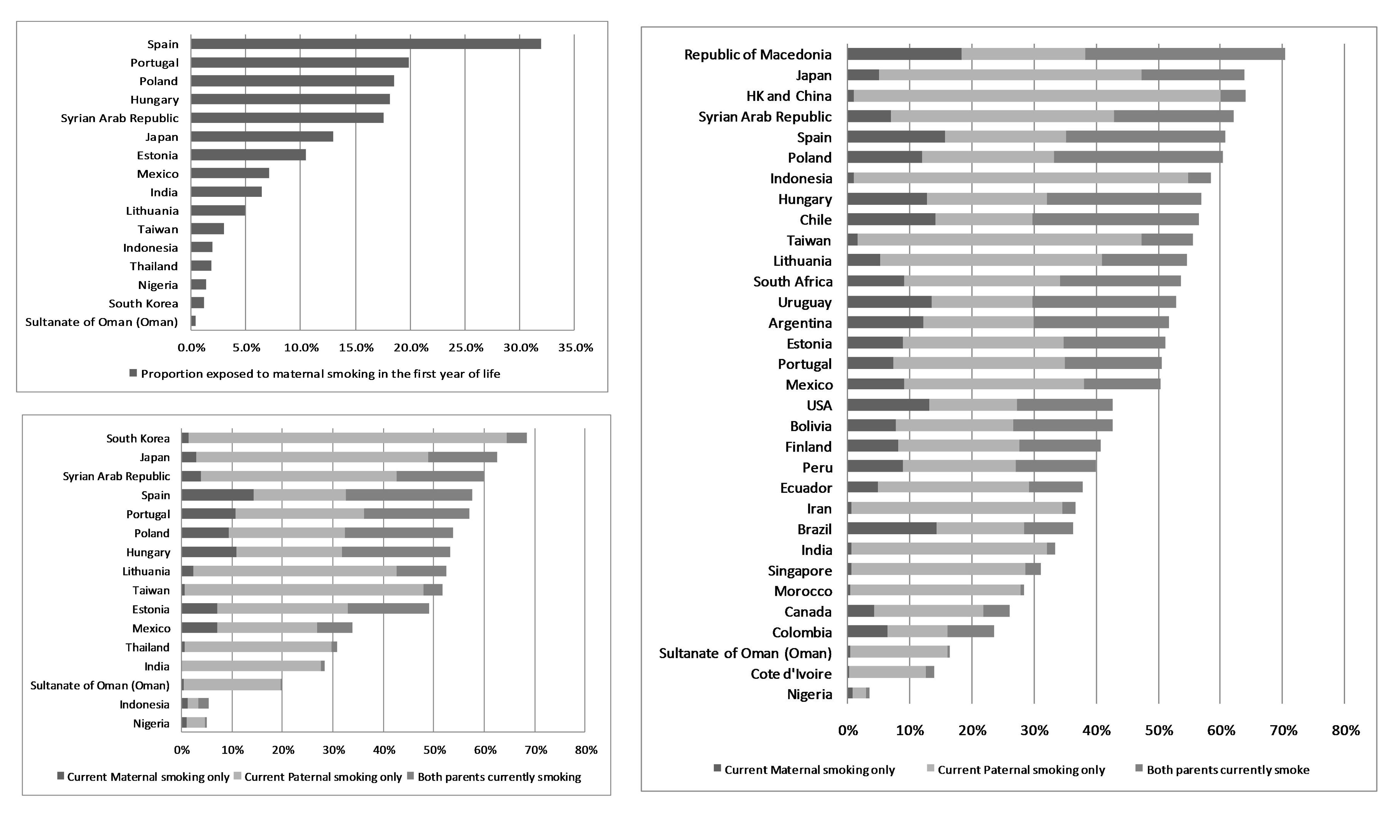

Supplement: Additional file 4: Figure S2. — Reported exposure of study subjects to parental smoking. Panel (a) shows the proportion of 6–7 year olds exposed maternal smoking in their first year of life, panel (b) shows the proportion of 6–7 year olds exposed to any current parental smoking, and panel (c) shows the proportion of adolescents exposed to any current parental smokingii. (TIFF 8254 kb) [file 12887_2015_538_MOESM4_ESM.tiff]
