# Supplementary material for: Maternal post-natal tobacco use and current parental tobacco use is associated with higher body mass index in children and adolescents: an international cross-sectional study
Source: BMC Pediatr. 2015 Dec 24;15:220. doi: 10.1186/s12887-015-0538-x (PMC4690230; doi:10.1186/s12887-015-0538-x)
Supplement: Additional file 2: Table S1. — basic characteristics of contributing centres for 6–7 year old children, including association between parental smoking and BMI (+/- kg/m2, (SE)) of participants in each centre. (DOC 73 kb) [file 12887_2015_538_MOESM2_ESM.doc]

**Additional file 2: Table S1 basic characteristics of contributing centres for 6-7 year old children, including association between parental smoking and BMI (+/- kg/m^2^, (SE)) of participants in each centre.**

| **Centre** | **GNI Category** | **N** | **Females (%)** | **Mother smoked 1st year of life (%)** | **Mother currently smokes (%)** | **Father currently smokes (%)** | **Age (mean years)** | **BMI (Kg/m^2^)** | **Mother smoked 1st year of life** | **Mother currently smokes** | **Father currently smokes** |
| --- | --- | --- | --- | --- | --- | --- | --- | --- | --- | --- | --- |
| **Cartagena, Spain** | High | 2282 | 50 | 34 | 45 | 49 | 6.6 | 17.5 | 0.14 (0.14) | 0.12 (0.13) | 0.44 (0.13) |
| **Valencia, Spain** | High | 2488 | 51 | 35 | 43 | 45 | 6.6 | 16.9 | 0.08 (0.11) | 0.23 (0.11) | 0.17 (0.11) |
| **Madrid, Spain** | High | 1863 | 50 | 32 | 40 | 45 | 6.5 | 16.9 | 0.28 (0.14) | 0.10 (0.13) | 0.16 (0.13) |
| **Asturias, Spain** | High | 2808 | 50 | 35 | 40 | 41 | 6.6 | 16.9 | 0.30 (0.10) | 0.18 (0.10) | 0.23 (0.09) |
| **Bilbao, Spain** | High | 2819 | 52 | 35 | 39 | 43 | 6.5 | 16.9 | 0.22 (0.09) | 0.15 (0.09) | 0.16 (0.09) |
| **A Coruña, Spain** | High | 2334 | 51 | 30 | 37 | 42 | 6.5 | 16.9 | 0.27 (0.11) | 0.30 (0.10) | 0.32 (0.10) |
| **Barcelona, Spain** | High | 2764 | 46 | 24 | 34 | 40 | 6.6 | 16 | 0.48 (0.10) | 0.29 (0.08) | 0.13 (0.08) |
| **Poznan, Poland** | High | 1712 | 49 | 20 | 33 | 46 | 7.2 | 16.1 | 0.18 (0.15) | 0.10 (0.13) | -0.05 (0.12) |
| **Kraków, Poland** | High | 2214 | 50 | 18 | 30 | 43 | 6.9 | 15.8 | 0.08 (0.14) | 0.26 (0.11) | 0.27 (0.10) |
| **Svábhegy, Hungary** | High | 2387 | 51 | 18 | 33 | 42 | 6.9 | 16.1 | 0.36 (0.15) | 0.36 (0.11) | 0.27 (0.10) |
| **Portimao, Portugal** | High | 990 | 48 | 20 | 32 | 47 | 6.4 | 16.3 | -0.06 (0.23) | 0.16 (0.19) | 0.15 (0.18) |
| **Tallinn, Estonia** | High | 1955 | 49 | 11 | 24 | 42 | 6.7 | 15.9 | 0.18 (0.15) | 0.20 (0.11) | 0.27 (0.09) |
| **Paysandú, Uruguay** | High | 1462 | 52 | 17 | 24 |  | 6.5 | 16.6 | 0.25 (0.20) | 0.26 (0.17) |  |
| **Ciudad de México (1), Mexico** | High | 2383 | 50 | 11 | 19 | 28 | 6.6 | 17 | -0.13 (0.22) | 0.05 (0.18) | -0.01 (0.15) |
| **Toluca, Mexico** | High | 3166 | 52 | 6 | 13 | 26 | 6.5 | 16.5 | 0.07 (0.16) | -0.05 (0.11) | 0.06 (0.10) |
| **Ciudad de México (3), Mexico** | High | 3406 | 51 | 6 | 12 | 26 | 6.5 | 16.4 | 0.17 (0.18) | 0.22 (0.14) | 0.13 (0.10) |
| **Antwerp, Belgium** | High | 3743 | 50 |  | 18 | 24 | 6.9 | 15.7 |  | 0.29 (0.10) | 0.37 (0.09) |
| **Fukuoka, Japan** | High | 2495 | 49 | 14 | 18 | 60 | 6.6 | 15.5 | 0.23 (0.12) | 0.18 (0.11) | 0.01 (0.08) |
| **Kaunas, Lithuania** | High | 1997 | 50 | 5 | 13 | 50 | 7 | 15.8 | -0.10 (0.19) | 0.21 (0.14) | 0.14 (0.09) |
| **Provincial Korea, South Korea** | High | 3458 | 47 | 1 | 6 | 68 | 6.8 | 16.3 | 0.31 (0.32) | 0.19 (0.18) | 0.20 (0.08) |
| **Seoul, South Korea** | High | 1468 | 48 | 1 | 5 | 65 | 6.9 | 16.6 | -0.43 (0.79) | -0.17 (0.29) | 0.02 (0.13) |
| **Taipei, Taiwan** | High | 3513 | 47 | 3 | 5 | 49 | 6.3 | 16.3 | 0.18 (0.27) | 0.18 (0.21) | 0.40 (0.08) |
| **Taoyuan, Taiwan** | High | 2378 | 49 | 3 | 4 | 54 | 6.8 | 16.5 | -0.36 (0.29) | -0.34 (0.28) | 0.15 (0.11) |
| **Al-Khod, Sultanate Of Oman** | High | 3903 | 49 | 0.50 | 0.60 | 20 | 6.7 | 14.3 | -0.64 (0.27) | -0.20 (0.33) | 0.21 (0.07) |
| **Centre** | **GNI Category** | **N** | **Females** | **Mother smoked 1st year of life** | **Mother currently smokes** | **Father currently smokes** | **Age (mean years)** | **BMI (Kg/m^2^)** | **Mother smoked 1st year of life** | **Mother currently smokes** | **Father currently smokes** |
| **Lattakia, Syria** | Low | 2309 | 54 | 19 | 23 | 56 | 6.2 | 15.6 | 0.17 (0.11) | 0.13 (0.11) | -0.00 (0.09) |
| **Tartous, Syria** | Low | 2676 | 49 | 16 | 20 | 56 | 6.7 | 15.6 | -0.03 (0.11) | 0.04 (0.11) | -0.05 (0.09) |
| **Bandung, Indonesia** | Low | 2352 | 49 | 2 | 3 | 4 | 6.6 | 14.2 | 0.69 (0.50) | 0.27 (0.32) | 0.02 (0.30) |
| **Bangkok, Thailand** | Low | 2852 | 43 | 2 | 2 | 31 | 6.4 | 16.7 | 0.63 (0.55) | -0.07 (0.52) | -0.16 (0.13) |
| **Chiang Mai, Thailand** | Low | 3014 | 52 | 2 | 2 | 30 | 6.2 | 15.8 | -0.70 (0.25) | -0.41 (0.29) | -0.28 (0.11) |
| **Ibadan, Nigeria** | Low | 2284 | 50 | 1 | 1 | 4 | 6.5 | 15 | 0.19 (0.38) | 0.56 (0.40) | 0.13 (0.23) |
| **Mumbai (29), India** | Low | 1717 | 54 | 6 | 0.90 | 28 | 6.6 | 13.5 | -0.03 (0.11) | -0.24 (0.21) | 0.06 (0.07) |

Countries are sorted into high and low GNI categories and then by percent current maternal smoking. Blank cells mean data was not collected for that variable in that centre.
