# Supplementary material for: Maternal post-natal tobacco use and current parental tobacco use is associated with higher body mass index in children and adolescents: an international cross-sectional study
Source: BMC Pediatr. 2015 Dec 24;15:220. doi: 10.1186/s12887-015-0538-x (PMC4690230; doi:10.1186/s12887-015-0538-x)
Supplement: Additional file 3: Table S2. — basic characteristics of contributing centres for adolescents, including association between parental smoking and BMI (+/- kg/m2, (SE)) of participants in each centre. (DOC 122 kb) [file 12887_2015_538_MOESM3_ESM.doc]

**Additional file 3: Table S2 basic characteristics of contributing centres for adolescents, including association between parental smoking and BMI (+/- kg/m^2^, (SE)) of participants in each centre.**

| **Centre** | **GNI Category** | **N** | **Females (%)** | **Mother currently smokes (%)** | **Father currently smokes (%)** | **Age (Mean years)** | **BMI (kg/m^2^)** | **Mother currently smokes** | **Father currently smokes** |
| --- | --- | --- | --- | --- | --- | --- | --- | --- | --- |
| **South Santiago, Chile** | High | 2630 | 56 | 47 | 48 | 13.5 | 20.5 | +0.23 (0.12) | +0.08 (0.12) |
| **Calama, Chile** | High | 1274 | 51 | 40 | 44 | 13.5 | 20 | +0.32 (0.18) | -0.16 (0.17) |
| **Chiloe, Chile** | High | 2834 | 52 | 36 | 36 | 13.5 | 21.2 | +0.11 (0.12) | +0.05 (0.12) |
| **Cartagena, Spain** | High | 3431 | 49 | 46 | 50 | 14 | 20.7 | +0.11 (0.11) | +0.25 (0.11) |
| **Valladolid, Spain** | High | 2726 | 47 | 43 | 46 | 13.5 | 19.9 | +0.20 (0.11) | +0.28 (0.10) |
| **Valencia, Spain** | High | 2799 | 46 | 43 | 45 | 13.6 | 19.9 | +0.16 (0.10) | +0.40 (0.10) |
| **Madrid, Spain** | High | 2186 | 42 | 42 | 50 | 13.4 | 19.6 | +0.22 (0.12) | +0.16 (0.12) |
| **Asturias, Spain** | High | 3595 | 50 | 42 | 46 | 13.9 | 20.3 | +0.27 (0.10) | +0.29 (0.10) |
| **Bilbao, Spain** | High | 2825 | 50 | 41 | 41 | 13.7 | 19.9 | +0.15 (0.10) | +0.13 (0.10) |
| **A Coruña, Spain** | High | 2510 | 47 | 39 | 41 | 13.5 | 19.9 | +0.38 (0.11) | +0.23 (0.11) |
| **San Sebastián, Spain** | High | 1110 | 47 | 38 | 37 | 13.4 | 19.9 | +0.12 (0.16) | +0.55 (0.16) |
| **Barcelona, Spain** | High | 2886 | 52 | 35 | 44 | 13.6 | 20.4 | +0.08 (0.10) | +0.30 (0.10) |
| **Montevideo, Uruguay** | High | 2899 | 51 | 44 | 41 | 13.6 | 20.2 | +0.11 (0.11) | +0.21 (0.11) |
| **Paysandú, Uruguay** | High | 1697 | 54 | 26 | 36 | 13.5 | 20 | +0.37 (0.16) | +0.24 (0.15) |
| **Poznan, Poland** | High | 1706 | 49 | 39 | 46 | 13.3 | 18.9 | +0.63 (0.13) | +0.37 (0.13) |
| **Kraków, Poland** | High | 2226 | 52 | 39 | 50 | 13.5 | 18.9 | +0.35 (0.10) | +0.23 (0.10) |
| **Svábhegy, Hungary** | High | 4102 | 51 | 38 | 44 | 13.5 | 19.5 | +0.50 (0.10) | +0.31 (0.09) |
| **Córdoba, Argentina** | High | 2966 | 43 | 37 | 39 | 13.5 | 19.2 | -0.03 (0.10) | -0.03 (0.10) |
| **Salta, Argentina** | High | 2419 | 55 | 31 | 40 | 13.6 | 19.6 | +0.04 (0.13) | -0.38 (0.12) |
| **Bay of Plenty, New Zealand** | High | 1578 | 50 | 35 |  | 14 | 22.5 | +1.37 (0.22) |  |
| **Nelson, New Zealand** | High | 1853 | 45 | 21 |  | 13.8 | 20.5 | +0.69 (0.20) |  |
| **Christchurch, New Zealand** | High | 2425 | 56 | 20 |  | 13.5 | 20.3 | +1.17 (0.18) |  |
| **Sarasota, Usa** | High | 922 | 52 | 29 | 29 | 13.6 | 20.7 | +1.01 (0.25) | +0.61 (0.26) |
| **Portimao, Portugal** | High | 1061 | 50 | 29 | 44 | 13.5 | 20.1 | -0.07 (0.21) | +0.08 (0.19) |
| **Centre** | **GNI Category** | **N** | **Females** | **Mother currently smokes (%)** | **Father currently smokes (%)** | **Age (Mean years)** | **BMI (kg/m^2^)** | **Mother currently smokes** | **Father currently smokes** |
| **Lisbon, Portugal** | High | 2367 | 54 | 26 | 42 | 13.4 | 20.2 | +0.21 (0.15) | +0.24 (0.13) |
| **Porto, Portugal** | High | 2770 | 51 | 23 | 44 | 13.5 | 20.1 | -0.17 (0.12) | +0.09 (0.11) |
| **Funchal, Portugal** | High | 2367 | 53 | 19 | 42 | 13.5 | 20.5 | +0.07 (0.19) | +0.15 (0.14) |
| **Cape Town, South Africa** | High | 4652 | 61 | 29 | 44 | 13.4 | 19.7 | -0.19 (0.11) | -0.34 (0.10) |
| **Tallinn, Estonia** | High | 3241 | 52 | 26 | 42 | 13.5 | 19 | +0.23 (0.10) | +0.17 (0.09) |
| **Ciudad de México (1), Mexico** | High | 3783 | 48 | 24 | 36 | 13.4 | 21.3 | +0.36 (0.13) | +0.16 (0.11) |
| **Ciudad de México (4), Mexico** | High | 2582 | 55 | 24 | 33 | 13.5 | 21.2 | +0.20 (0.17) | -0.12 (0.14) |
| **Villahermosa, Mexico** | High | 2405 | 50 | 15 | 20 | 13.3 | 20.9 | -0.22 (0.21) | +0.21 (0.20) |
| **Toluca, Mexico** | High | 2958 | 52 | 21 | 72 | 13.3 | 20.1 | +0.32 (0.14) | +0.03 (0.12) |
| **Fukuoka, Japan** | High | 2033 | 45 | 22 | 59 | 13.6 | 18.7 | +0.50 (0.14) | +0.24 (0.11) |
| **Kuopio County, Finland** | High | 2809 | 49 | 21 | 32 | 13.5 | 20 | +0.59 (0.14) | +0.33 (0.12) |
| **Kaunas, Lithuania** | High | 2514 | 51 | 20 | 49 | 13.5 | 18.5 | +0.36 (0.12) | +0.16 (0.10) |
| **Seoul, South Korea** | High | 2653 | 38 | 11 |  | 13.5 | 19.6 | +0.12 (0.19) |  |
| **Provincial Korea, South Korea** | High | 6779 | 47 | 10 |  | 13.5 | 19.5 | 0.00 (0.12) |  |
| **Taipei, Taiwan** | High | 5868 | 49 | 11 | 53 | 13.3 | 20.1 | +0.36 (0.15) | +0.33 (0.09) |
| **Taoyuan, Taiwan** | High | 2897 | 50 | 8 | 56 | 13.8 | 20.3 | +0.80 (0.29) | +0.35 (0.13) |
| **Vancouver, Canada** | High | 2483 | 47 | 9 | 22 | 13.5 | 20.1 | +0.85 (0.23) | +0.26 (0.17) |
| **Singapore, Singapore** | High | 3810 | 59 | 3 | 30 | 13.3 | 19.2 | +0.47 (0.36) | -0.10 (0.12) |
| **Al-Khod, Sultanate Of Oman** | High | 3126 | 51 | 1 | 16 | 13.5 | 19.3 | +0.21 (0.68) | +0.64 (0.20) |
| **Skopje, Republic Of Macedonia** | Low | 2869 | 49 | 51 | 52 | 13.5 | 19.9 | +0.62 (0.10) | +0.32 (0.11) |
| **Lattakia, Syria** | Low | 2946 | 65 | 33 | 57 | 13.3 | 19.6 | +0.19 (0.12) | -0.09 (0.11) |
| **Tartous, Syria** | Low | 2879 | 53 | 28 | 53 | 13.3 | 19.3 | +0.27 (0.14) | +0.18 (0.12) |
| **Aleppo, Syria** | Low | 2763 | 50 | 18 | 56 | 12.9 | 19.3 | +0.27 (0.15) | -0.00 (0.11) |
| **Nova Iguaçu, Brasil** | Low | 3018 | 51 | 24 | 23 | 13.5 | 19.5 | +0.04 (0.14) | -0.08 (0.14) |
| **Vitória da Conquista, Brasil** | Low | 1155 | 53 | 15 | 19 | 13.5 | 19.2 | +0.10 (0.24) | +0.11 (0.24) |
| **Centre** | **GNI Category** | **N** | **Females** | **Mother currently smokes (%)** | **Father currently smokes (%)** | **Age (Mean years)** | **BMI (kg/m^2^)** | **Mother currently smokes** | **Father currently smokes** |
| **Santa Cruz, Bolivia** | Low | 2754 | 52 | 24 | 35 | 13.4 | 20.5 | -0.13 (0.15) | -0.00 (0.14) |
| **Lima, Peru** | Low | 2588 | 35 | 22 | 31 | 13.5 | 19.9 | +0.10 (0.13) | -0.01 (0.12) |
| **Suva, Fiji** | Low | 3035 | 54 | 19 |  | 13.3 | 20.1 | +0.67 (0.16) |  |
| **Barranquilla, Colombia** | Low | 2398 | 57 | 14 | 17 | 13.5 | 19.1 | +0.28 (0.19) | +0.03 (0.18) |
| **Guayaquil, Ecuador** | Low | 3006 | 51 | 14 | 33 | 13.4 | 20.9 | +0.53 (0.18) | -0.09 (0.13) |
| **Beijing, China** | Low | 3308 | 49 | 9 | 69 | 13.5 | 20.2 | +0.38 (0.28) | +0.09 (0.16) |
| **Tong Zhou, China** | Low | 3440 | 50 | 7 | 74 | 13.8 | 19.5 | +0.31 (0.22) | +0.01 (0.13) |
| **Guangzhou, China** | Low | 3273 | 52 | 4 | 67 | 13.4 | 18.4 | -0.25 (0.25) | +0.14 (0.11) |
| **Wulumuqi(9), China** | Low | 3250 | 49 | 3 | 67 | 13.1 | 18.3 | +0.06 (0.35) | +0.10 (0.11) |
| **Hong Kong, China** | Low | 2914 | 50 | 3 | 32 | 13.8 | 19.5 | +0.29 (0.33) | +0.46 (0.13) |
| **Tibet, China** | Low | 2806 | 51 | 4 | 62 | 13.5 | 18.8 | -0.25 (0.20) | -0.14 (0.10) |
| **Bandung, Indonesia** | Low | 2763 | 52 | 9 | 68 | 13.1 | 17.4 | +0.34 (0.20) | -0.40 (0.12) |
| **Semarang, Indonesia** | Low | 2242 | 55 | 2 | 56 | 13.5 | 17.9 | +0.01 (0.38) | -0.01 (0.12) |
| **Bali, Indonesia** | Low | 2466 | 49 | 2 | 47 | 13.2 | 17.7 | +0.71 (0.35) | -0.03 (0.11) |
| **Tehran, Iran** | Low | 2248 | 43 | 4 | 37 | 13.2 | 18.9 | +0.48 (0.40) | -0.14 (0.15) |
| **Rasht, Iran** | Low | 2161 | 47 | 1 | 35 | 13.3 | 19.3 | +0.54 (0.45) | +0.19 (0.17) |
| **Casablanca, Morocco** | Low | 1647 | 48 | 2 | 33 | 13.3 | 18.8 | +0.39 (0.60) | +0.06 (0.18) |
| **Boulmene, Morocco** | Low | 1217 | 41 | 0.70 | 22 | 13.3 | 17.3 | -0.53 (0.52) | +0.20 (0.16) |
| **Marrakech, Morocco** | Low | 1653 | 58 | 0.50 | 28 | 13.4 | 19.1 | +0.50 (1.32) | +0.04 (0.16) |
| **Ibadan, Nigeria** | Low | 2904 | 44 | 2 | 3 | 13.3 | 17.9 | -0.08 (0.42) | +0.42 (0.29) |
| **Urban Cote d Ivoire, Cote D'ivoire** | Low | 3241 | 40 | 2 | 14 | 13.1 | 21.2 | -0.16 (0.34) | +0.01 (0.12) |
| **New Delhi (7), India** | Low | 3256 | 65 | 2 | 29 | 13.5 | 20.1 | -0.71 (0.47) | -0.30 (0.15) |
| **Mumbai (29), India** | Low | 1770 | 52 | 1 | 39 | 13.3 | 15.8 | +0.49 (0.40) | -0.13 (0.11) |

Countries are sorted into high and low GNI categories and then by percent current maternal smoking. Blank cells mean data was not collected for that variable in that centre.
